# Supplementary material for: A successional shift enhances stability in ant symbiont communities
Source: Commun Biol. 2024 May 27;7:645. doi: 10.1038/s42003-024-06305-3 (PMC11130137; doi:10.1038/s42003-024-06305-3)
Supplement: Supplementary file 1 — Supplemental material [file 42003_2024_6305_MOESM1_ESM.docx]

**Supplementary Information:**

A successional shift enhances stability in ant symbiont communities

Thomas Parmentier, Dries Bonte, Frederik De Laender

## Supplementary tables

**Table S1.** Summary measures of model selection and model fit. WAIC stands for WidelyApplicable Information Criterion, R^2^ gives the explanatory power of each model. Predictors: con = connectivity, can = canopy openness, quadratic terms are indicated with ^2^.

| Model | Predictors | WAIC | R^2^ |
| --- | --- | --- | --- |
| 1 | age+con+moisture+moisture^2^+pH+can | 511.3 | 0.51 |
| 2 | age+con+moisture+pH+pH^2^+can | 611.6 | 0.47 |
| 3 | age+con+moisture+moisture^2^+pH+pH^2^+can | 502.9 | 0.47 |
| 4 | age+con+moisture+moisture^2^+pH+pH^2^+can+can^2^ | 673.5 | 0.49 |
| 5 | age+con+moisture+moisture^2^+pH+can+can^2^ | 490.7 | 0.55 |
| 6 | age+con+moisture+pH+pH^2^+can+can^2^ | 359.4 | 0.54 |
| 7 | age+con+moisture+pH+can+can^2^ | 339.3 | 0.55 |
| 8 | age+con+moisture+pH+can | 340.2 | 0.55 |

Model 7 was retained based on the lowest WAIC value. This model also had the highest R^2^. The MCMC convergence of model 7 was satisfactory. The trace plots displayed an irregular pattern with similar running means across all chains. The potential scale reduction factors were smaller than 1.1 and the effective sample size was close to the actual sample size of 4000 (each of the 4 chains has 1000 samples).

The predictive power of the retained model (R^2^ = 0.10), was lower than the explanatory power (R^2^ = 0.55). This relatively low predictive power may be related to a relatively small number of sampled nests (N = 51). Since our research places a greater emphasis on inference rather than prediction, we have confidence in the robust support for our conclusions. This is evident by the strong statistical support of relevant parameter estimates, despite the low predictive power of the model.

**Table S2**. General adjacency matrix *A*, representing overall food web relationships between 16 functional symbiont groups. Rows are resources and columns are consumers. An element *a_ij_* = 1 signifies that *i* consumed *j*, when *a_ij_* = 0 no trophic interaction is assumed between *i* and *j*.

|  | spider_P_ | rove beetle_P_ | rove beetle_SP1_ | rove beetle_SP2_ | rove beetle_S_ | beetle_D_ | beetle_B_ | springtail_D_ | isopod_D_ | fac armoured_D_ | fac pred_P1_ | fac pred_P2_ | fac prey_D_ | fac rove beetle_S_ | fac mite_D_ | fac mite_P_ | ant brood | nest food |
| --- | --- | --- | --- | --- | --- | --- | --- | --- | --- | --- | --- | --- | --- | --- | --- | --- | --- | --- |
| spider_P_ | NaN | 0 | 1 | 0 | 0 | 0 | 0 | 0 | 0 | 0 | 1 | 0 | 0 | 0 | 0 | 0 | 0 | 0 |
| rove beetle_P_ | 1 | NaN | 1 | 1 | 0 | 0 | 0 | 0 | 0 | 0 | 1 | 0 | 0 | 0 | 0 | 0 | 0 | 0 |
| rove beetle_SP1_ | 0 | 0 | NaN | 0 | 0 | 0 | 0 | 0 | 0 | 0 | 0 | 0 | 0 | 0 | 0 | 0 | 0 | 0 |
| rove beetle_SP2_ | 1 | 0 | 1 | NaN | 0 | 0 | 0 | 0 | 0 | 0 | 1 | 0 | 0 | 0 | 0 | 0 | 0 | 0 |
| rove beetle_S_ | 1 | 0 | 1 | 1 | NaN | 0 | 0 | 0 | 0 | 0 | 1 | 0 | 0 | 0 | 0 | 0 | 0 | 0 |
| beetle_D_ | 1 | 0 | 1 | 1 | 0 | NaN | 0 | 0 | 0 | 0 | 1 | 0 | 0 | 0 | 0 | 0 | 0 | 0 |
| beetle_B_ | 0 | 0 | 0 | 0 | 0 | 0 | NaN | 0 | 0 | 0 | 0 | 0 | 0 | 0 | 0 | 0 | 0 | 0 |
| springtail_D_ | 1 | 1 | 0 | 0 | 0 | 0 | 0 | NaN | 0 | 0 | 1 | 1 | 0 | 0 | 0 | 1 | 0 | 0 |
| isopod_D_ | 0 | 0 | 1 | 0 | 0 | 0 | 0 | 0 | NaN | 0 | 1 | 0 | 0 | 0 | 0 | 0 | 0 | 0 |
| fac armoured_D_ | 0 | 0 | 0 | 0 | 0 | 0 | 0 | 0 | 0 | NaN | 0 | 0 | 0 | 0 | 0 | 0 | 0 | 0 |
| fac pred_P1_ | 0 | 0 | 0 | 0 | 0 | 0 | 0 | 0 | 0 | 0 | NaN | 0 | 0 | 0 | 0 | 0 | 0 | 0 |
| fac pred_P2_ | 1 | 1 | 1 | 0 | 0 | 0 | 0 | 0 | 0 | 0 | 1 | NaN | 0 | 0 | 0 | 0 | 0 | 0 |
| fac prey_D_ | 1 | 1 | 1 | 1 | 0 | 0 | 0 | 0 | 0 | 0 | 1 | 1 | NaN | 0 | 0 | 1 | 0 | 0 |
| fac rove beetle_S_ | 1 | 0 | 1 | 1 | 0 | 0 | 0 | 0 | 0 | 0 | 1 | 0 | 0 | NaN | 0 | 0 | 0 | 0 |
| fac mite_D_ | 1 | 1 | 1 | 1 | 0 | 0 | 0 | 0 | 0 | 0 | 1 | 1 | 0 | 1 | NaN | 1 | 0 | 0 |
| fac mite_P_ | 1 | 1 | 1 | 1 | 0 | 0 | 0 | 0 | 0 | 0 | 1 | 1 | 0 | 1 | 0 | NaN | 0 | 0 |
| ant brood | 0 | 0 | 0 | 1 | 1 | 1 | 1 | 0 | 1 | 0 | 0 | 0 | 0 | 0 | 0 | 0 | 0 | 0 |
| nest food | 0 | 0 | 1 | 1 | 1 | 1 | 1 | 1 | 1 | 1 | 1 | 1 | 1 | 1 | 1 | 1 | 0 | 0 |

## Supplementary figures





**Figure S1.** Residual association in abundance shown for all pairs of functional groups after controlling for the predictors in the retained model 7. The functional groups are grouped in obligate and facultative red wood ant associates. Each matrix cell corresponds to a pair of functional groups and the gradient colours indicate the estimated association strength measured at correlation scale. Only associations with at least 95% posterior probability are displayed. Red colors depict positive residual associations among functional groups, no negative residual associations were detected


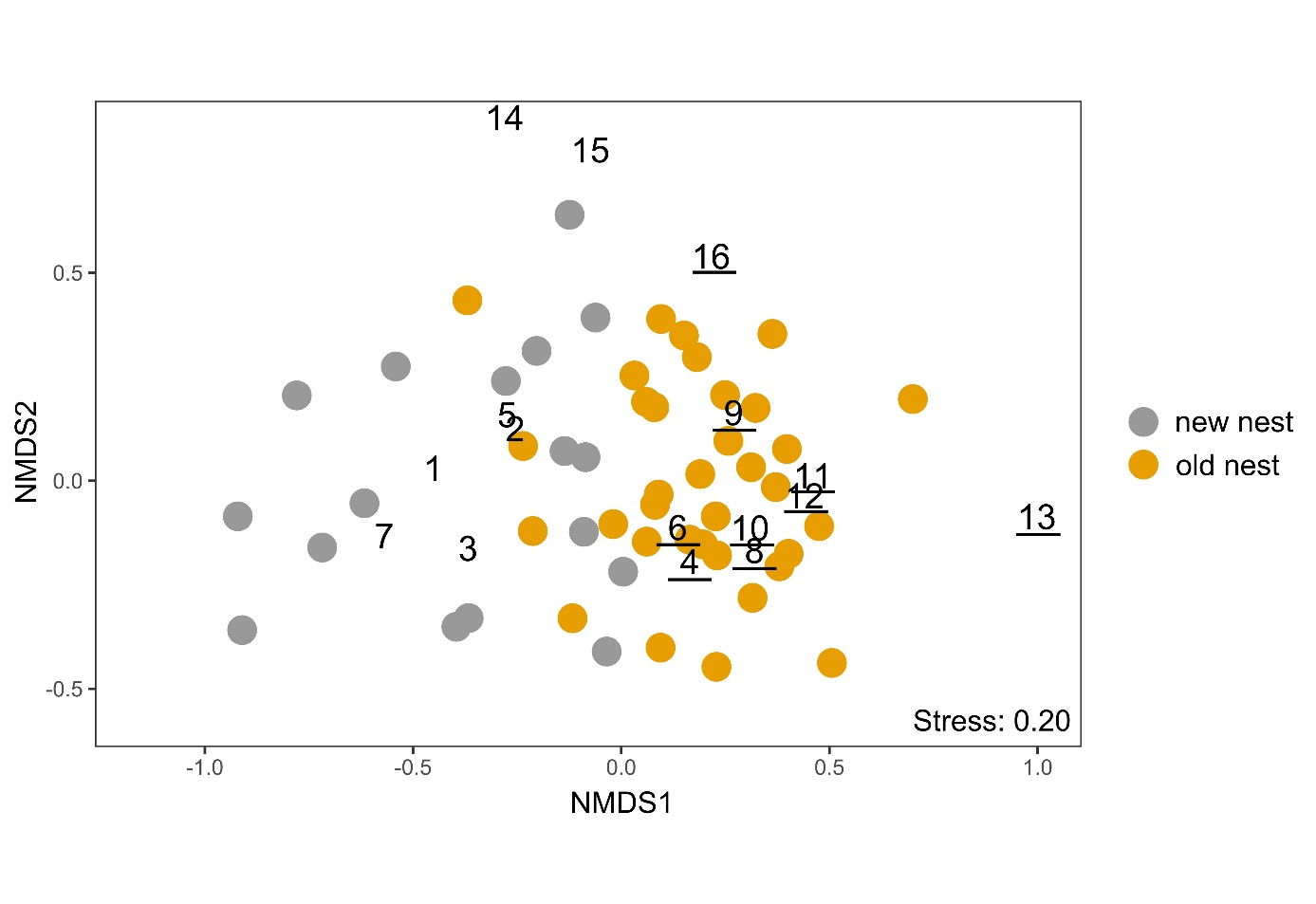


**Figure S2.** Multivariate analysis of the 51 nest-symbiont communities using Non-metric dimensional scaling. Communities in old nests are given in orange dots, those of new nests in gray dots. Number codes that are underlined refer to obligate functional groups found in the communities: 4) springtail_D_, 6) spider_P_ , 8) beetle_B_, 9) rove beetle_P_, 10) beetle_D_, 11) rove beetle_SP2_, 12) rove beetle_S_, 13) isopod_D_, 16) rove beetle_SP1_. Number codes that are not underlined represent facultative functional groups found in the communities: 1) fac mite_D_, 2) fac mite_P_, 3) fac prey_D_, 5) fac armoured_D_, 7) fac rove beetle_S_, 14) fac pred_P1_, 15) fac pred_P2_. Number codes of the functional groups are similar to those used in Fig. 3.


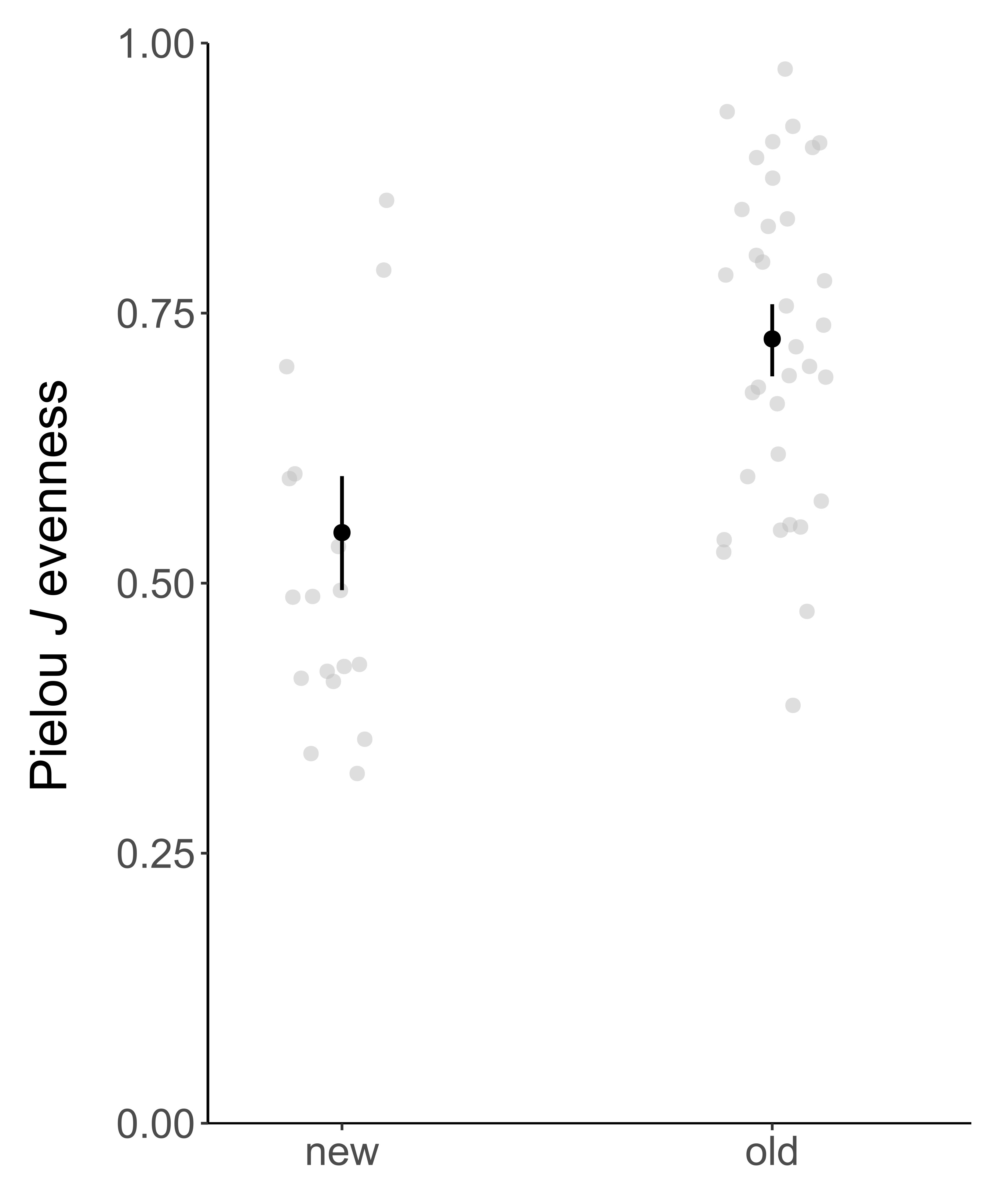


**Figure S3.** Evenness of symbiont communities in new and old nests as measured by the Pielou’s evenness index *J*, beta regression, P < 0.001.


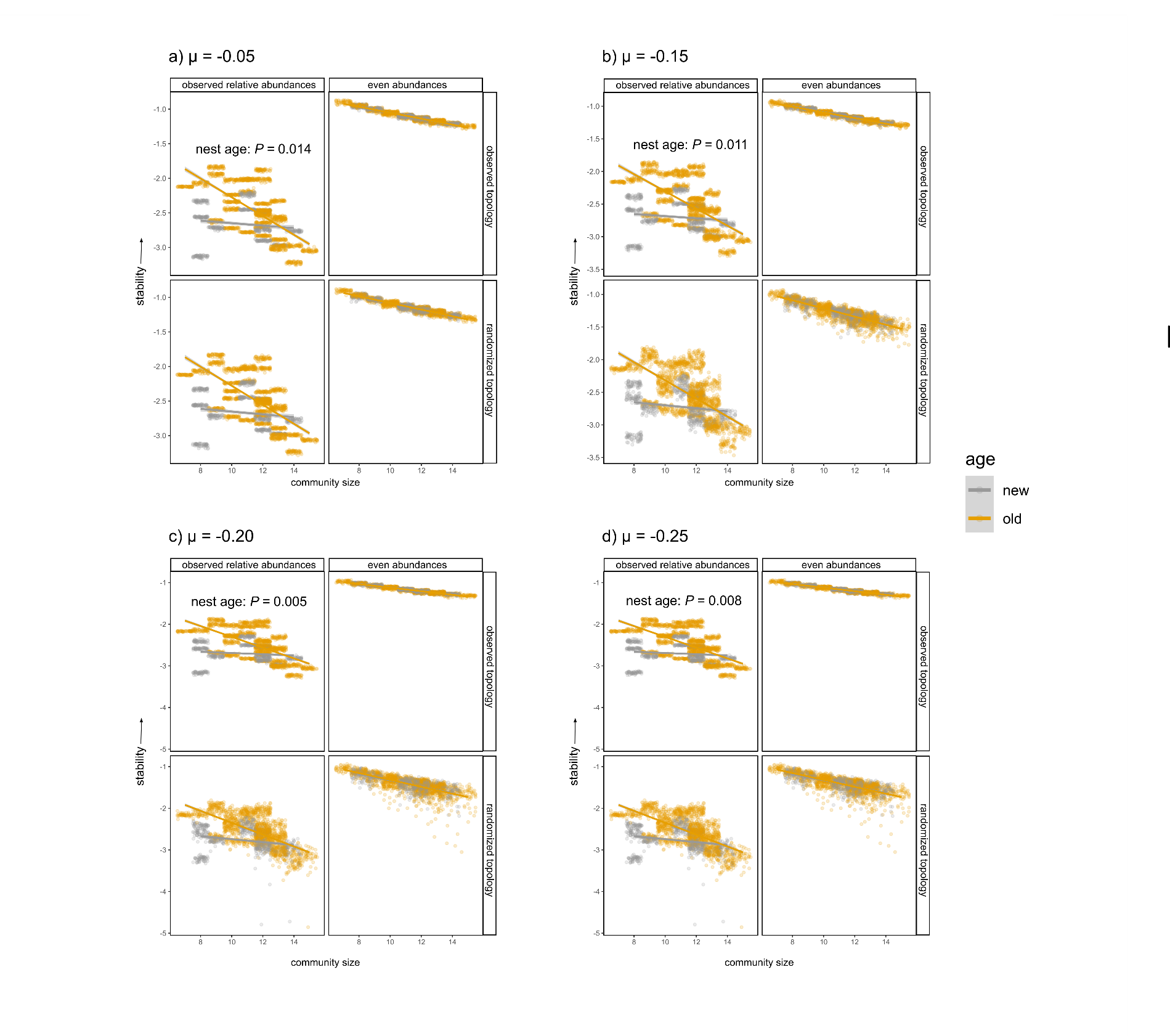


Figure S4. Prediction of local stability for symbiont food webs in old and new nests with increasing community size (= number of functional groups) in four scenarios and with different *µ*. Interaction coefficients were drawn from *a_ij_ ∼N*(*µ,σ*) with *µ* = -0.10 and *σ* = 0.05 in the main manuscript (Fig. 4). Here we conducted a sensitivity analysis with *a_ij_ ∼N*(*µ,σ*) and set *µ* to the values : a) *µ* = -0.05, b) *µ* = -0.15, c) µ = -0.20 and d) *µ* = -0.25. For the scenario with observed relative abundances and observed topology, older nests exhibited significantly greater stability compared to new nests, regardless of the parameter *µ* (corresponding *P-*values displayed on plot).
